# Stable functional structure despite high taxonomic variability across fungal communities in soils of old-growth montane forests

**DOI:** 10.1186/s40168-023-01650-7

**Published:** 2023-10-02

**Authors:** Qingchao Zeng, Annie Lebreton, Lucas Auer, Xiaowu Man, Liukun Jia, Gengshen Wang, Sai Gong, Vincent Lombard, Marc Buée, Gang Wu, Yucheng Dai, Zhuliang Yang, Francis M. Martin

**Affiliations:** 1 Beijing Advanced Innovation Center for Tree Breeding By Molecular Design, Beijing Forestry University, Beijing, 100083 China; 2 School of Ecology and Nature Conservation, Beijing Forestry University, Beijing, 100083 China; 3 Université de Lorraine, INRAE, UMR Interactions Arbres/Microorganismes, Centre INRAE Grand Est-Nancy, 54280 Champenoux, France; 4 INRAE, Aix-Marseille Université, Biodiversité et Biotechnologie Fongiques, 13009 Marseille, France; 5 CAS Key Laboratory for Plant Diversity and Biogeography of East Asia, Kunming Institute of Botany, Chinese Academy of Sciences, Kunming, 650201 Yunnan China; 6 Yunnan Key Laboratory for Fungal Diversity and Green Development, Kunming, 650201 Yunnan China; 7 Architecture Et Fonction Des Macromolécules Biologiques, CNRS, Aix-Marseille Université, UMR 7257, 13288 Marseille, France

**Keywords:** Fungal guilds, Functional traits, Forest soil, Metatranscriptomics, Organic Matter degradation, Yunnan

## Abstract

**Background:**

Major advances over the past decade in molecular ecology are providing access to soil fungal diversity in forest ecosystems worldwide, but the diverse functions and metabolic capabilities of this microbial community remain largely elusive. We conducted a field survey in montane old-growth broadleaved and conifer forests, to investigate the relationship between soil fungal diversity and functional genetic traits. To assess the extent to which variation in community composition was associated with dominant tree species (oak, spruce, and fir) and environmental variations in the old-growth forests in the Jade Dragon Snow Mountain in Yunnan Province, we applied rDNA metabarcoding. We also assessed fungal gene expression in soil using mRNA sequencing and specifically assessed the expression of genes related to organic matter decomposition and nutrient acquisition in ectomycorrhizal and saprotrophic fungi.

**Results:**

Our taxonomic profiling revealed striking shifts in the composition of the saprotrophic and ectomycorrhizal guilds among the oak-, fir-, and spruce-dominated forests. The core fungal microbiome comprised only ~ 20% of the total OTUs across all soil samples, although the overlap between conifer-associated communities was substantial. In contrast, seasonality and soil layer explained only a small proportion of the variation in community structure. However, despite their highly variable taxonomic composition, fungal guilds exhibited remarkably similar functional traits for growth-related and core metabolic pathways across forest associations, suggesting ecological redundancy. However, we found that the expression profiles of genes related to polysaccharide and protein degradation and nutrient transport notably varied between and within the fungal guilds, suggesting niche adaptation.

**Conclusions:**

Overall, our metatranscriptomic analyses revealed the functional potential of soil fungal communities in montane old-growth forests, including a suite of specialized genes and taxa involved in organic matter decomposition. By linking genes to ecological traits, this study provides insights into fungal adaptation strategies to biotic and environmental factors, and sheds light on the importance of understanding functional gene expression patterns in predicting ecosystem functioning.

Video Abstract

**Supplementary Information:**

The online version contains supplementary material available at 10.1186/s40168-023-01650-7.

## Introduction

Tropical and subtropical mountain ecosystems are experiencing particularly strong climate warming, which affects nutrient cycling and carbon (C) sequestration [1, 2]. Mountain plants have served as bioindicators of the impact of rapid environmental change [3]. Although the responses of mountain vegetation have been studied aboveground, there are parallel, unknown changes taking place belowground, where plant roots and their associated microbial communities form complex but largely unknown entangled networks. Global surveys have shown that soil microbial communities are affected by global changes including climate and seasonality, [4–9]. However, these studies have been limited to a few ecosystems and regions, primarily temperate and boreal forests. Despite growing evidence that soil microbes can play a critical role in regulating the dynamics of tropical and subtropical biomes, plant–microbiome interactions in such ecosystems remain largely understudied. These geographical gaps limit our ability to draw general inferences about the implications of the plant and soil microbiome, especially to comprehend how microbial effects on plant community dynamics vary with environmental cues. Therefore, it is critical to characterize soil microbial communities of (sub)tropical mountain forests to track the future effects of global changes on their composition, diversity, and functioning.

In forests, soil microbes play a key role in biogeochemical cycles, C storage, and rhizosphere processes [10, 11]. Soil fungi and bacteria release extracellular degrading enzymes such as carbohydrate-active enzymes (CAZymes) and proteases, which enable the decomposition of soil organic matter (SOM) compounds such as cellulose, lignin, chitin, and proteins [10]. Many soil fungi can form mutualistic mycorrhizal symbioses that promote plant growth through soil resource acquisition [12]. These complex processes are conducted by a diverse group of fungi that are interconnected through trophic networks and respond rapidly to changes in environmental conditions such as changes in plant associations [13, 14], litter accumulation and composition, precipitation, and drought [5, 7–9]. Thus, there is growing appreciation for the diversity of fungal communities and the environmental factors driving their assemblage. In contrast, it has proven challenging to assess the functions of these soil microbes in situ. Consequently, the genetic mechanisms behind resource acquisition and allocation by soil-borne and tree-associated fungi in response to environmental changes remain unclear, despite being critical for understanding ecosystem-scale nutrient dynamics. Therefore, we need to deepen our investigation beyond the sole distribution of taxa in fungal guilds by identifying key genes and delving into their expression characteristics. We need to determine how different fungal species adjust their levels of specific transcript expression with regard to the plant-driven microbiome state that they inhabit and to discover how gene expression stands in conjunction with soil, host, and climate features.

To date, the technical difficulties associated with extracting and sequencing eukaryotic mRNA sampled from soils and the lack of reference fungal genomes for mapping RNA reads have limited the capture of gene expression within entire fungal communities, hindering functional insights into ecological settings. Metatranscriptomics has been used to track gene expression in ectomycorrhizal roots collected in situ [15, 16] and in soil bacterial communities [17, 18]. Thus, the objective of the present study was to compare the distribution of the soil fungal community and its functional genetic traits in neighboring old-growth forests dominated by oaks, spruces, and firs on the Jade Dragon Snow Mountain (referred to as Yulongxueshan) in Yunnan Province, China. This subtropical montane region, located in the southern part of the Hengduan Mountains, is a major climatic border between the southwestern and southeastern monsoon systems of China and the northern Qinghai-Tibetan Plateau. It is recognized for its high plant biodiversity [19] and has accumulated exceptional biodiversity over long time scales as a result of historical and ecological factors, but is threatened by increased human activities [20].

We used high-throughput rDNA amplicon sequencing to identify the composition of soil fungal communities. We then assessed the extent to which spatial variation in the composition of microbial communities was associated with forest associations and environmental variations such as seasonality and soil features. The major goal of this soil microbial survey was to assess the gene expression of fungal communities using soil mRNA sequencing and RNA read mapping to reference fungal genomes. In addition to assessing total gene expression by fungal guilds, such as ectomycorrhizal and saprotrophic fungi, and changes in central metabolism and growth-related pathways, we specifically addressed the expression of genes which mediate nitrogen (N) cycling and organic matter (OM) decomposition. We implemented this experimental design to investigate the following hypotheses: (H1) similar to observations in temperate and boreal forests, the fungal community composition will display variations associated with different forest associations, with these responses being influenced by soil layer and season; (H2) gene expression profiles will differ between mycorrhizal and saprotrophic species due to their distinct genomic characteristics; and (H3) functional traits will reflect the diversity of fungal communities, given the well-established variability in nitrogen uptake and organic matter decomposition capacities across taxa [21, 22].

## Material and methods

### Site description

**Fig. 1:**
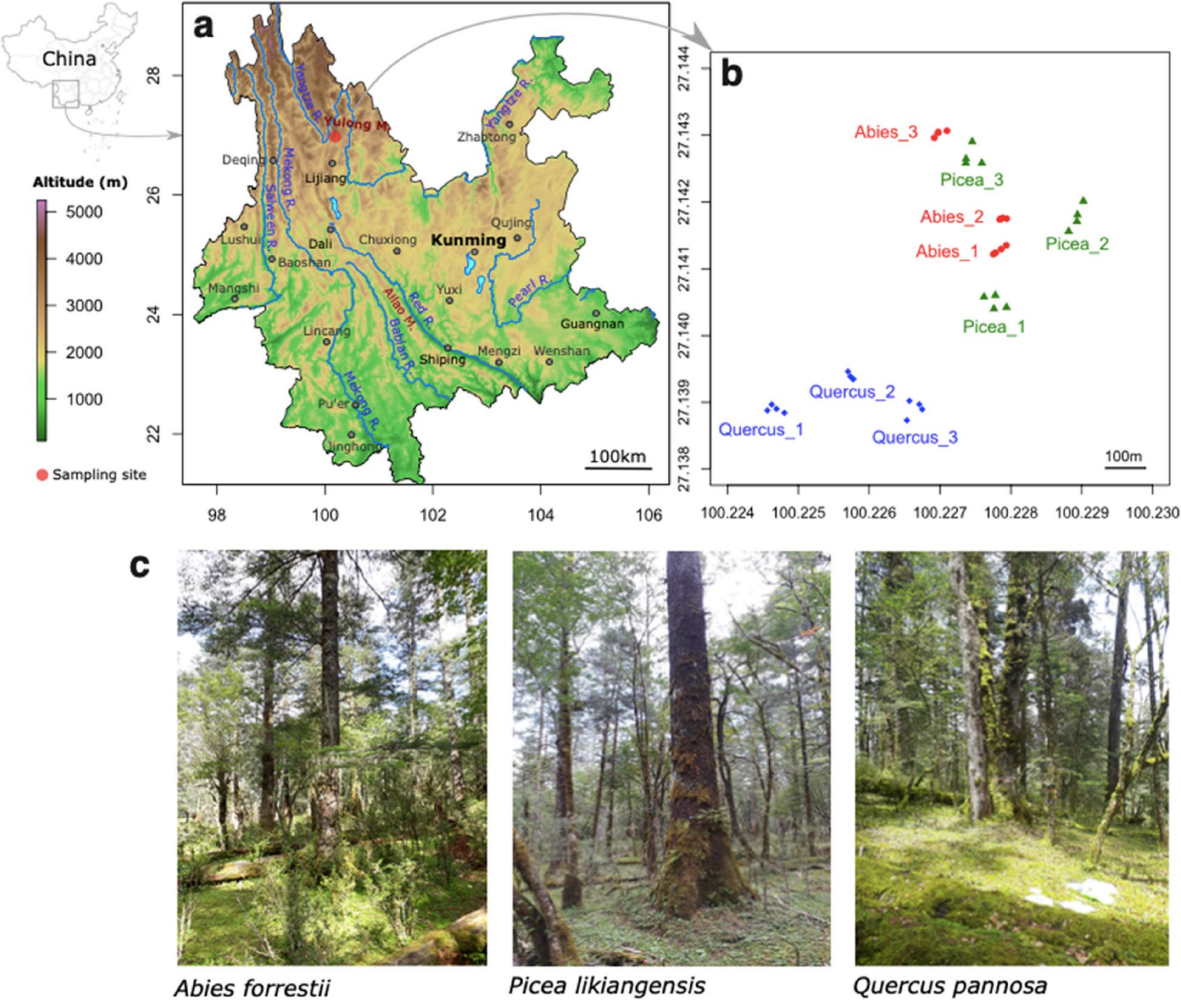
Location of the study site and forest plots in the Yulongxueshan, within the Hengduan Mountains, Yunnan Province. **a** The red dot on the Yunnan map indicates the Lijiang Forest Biodiversity National Observation and Research station in Yunshanping, at 3200 m asl. **b** Soil cores were collected from four clustered trees located in three plots per site, for each old-growth forest association. **c** Photographs of old-growth forests dominated by *Abies forrestii*, *Picea likiangensis*, and *Quercus pannosa (guyavifolia)*

A survey of soil fungal communities was conducted in the high-elevation forests of Yulongxueshan. This mountain range, located near Lijiang in the northwest of Yunnan Province, between the Yangtze and Mekong Rivers (Fig. 1), is known for its exceptional plant biodiversity. In the Lijiang Forest Biodiversity National Observation and Research Station (Yunshanping, 27°10′–27°40′ N, 100°10′–100°20′ E), a protected site covered by old-growth primary forests, we selected three plots located at ~ 3200 m, dominated by local species of evergreen oak (*Quercus pannosa (guyavifolia))*, Lijiang spruce (*Picea likiangensis*), and Forrest’s fir (*Abies forrestii*). The tree diameter at breast height (DBH) ranged from 155 to 345 cm, 103 to 396 cm, and 120 to 295 cm for selected oaks, spruces, and firs, respectively. The subcanopy layer contains *Gamblea ciliata*, *Acer pectinatum*, and *Padus brachypoda*, whereas the subtree layer is dominated by *Sorbus pteridophylla*, *Viburnum betulifolium*, *Rhododendron yunnanense*, and *Berberis levis* [23, 24]. The sample site is located in a cold-temperate climate with a mean annual temperature of 5.5 °C, a maximum temperature of 18.8 °C and a minimum temperature of − 20 °C (Supplementary Fig. S1). The annual precipitation is 1587 mm, the annual evaporation is 966 mm, and the relative humidity is 82%. The soil type was brown or dark brown perudic cambisols [25].

### Soil sampling and analysis

Sampling was conducted during the wet (monsoon) season in early August 2019 and 2020 and during the dry season in April 2020. Nine plots were sampled: three dominated by oaks, three by firs*,* and three by spruces. Within each plot, we sampled four individual trees separated by 5–15 m (Fig. 1), for a total of 36 trees. At each tree location, two soil cores, subsampled in near-surface soil (organic soils (OS); 0–5 cm depth) and deeper soils (organo-mineral soil (OM); 5–25 cm depth), were collected at 1 m (north and south) from the base of the trunk (144 soil cores in total for the wet and dry seasons) by using a steel helical soil sampler. Litter or plant detritus was discarded. Soil samples were immediately sieved through 5-mm meshes, homogenized, subsampled in sterile plastic tubes, and snap-frozen in dry ice. The all procedure took < 10 min. Samples were kept in dry ice chests to remain frozen in the field, and all samples were transported to the Kunming Institute of Botany, where they were stored at − 80 °C until further processing.

We evaluated soil nutrients and chemistry to gauge the changes across the three forest associations. The physicochemical characteristics (Supplementary Fig. S2) were measured according to the protocol described by Bao [26]. This includes pH, nitrogen (N), phosphorus (P), calcium (Ca), SOM, and CEC. Analyses were performed by Puluo Environmental Protection Technology Co., Ltd. (Guangzhou, China).

### DNA extraction, rDNA amplification, and rDNA sequencing

The DNA used for metabarcoding analyses was extracted and quality checked, as detailed by Zeng et al. [14]. In brief, total DNA was extracted from 50 mg of soil samples using the Qiagen DNeasy PowerSoil DNA Isolation Kit (Qiagen, Germany) according to the manufacturer’s protocol. DNA quality and quantity were assayed using a NanoDrop ONE spectrophotometer (Thermo Fisher Scientific, Waltham, MA, USA). DNA extractions were carried out in triplicates and PCR products were pooled to reduce extraction-related variance. Amplicon libraries were prepared in a single PCR reaction using Illumina- barcoded fungal-specific primers. Three libraries were prepared for metabarcoding using primers targeting rDNA ITS1 (ITS1-F, ITS2), rDNA ITS2 (ITS86F, ITS4), and rDNA 18S (M-HYJ-AMV4.5NF, M-HYJ-AMDGR), the latter of which is known to better detect Glomeromycotina (for a total of 431 samples). All PCR reactions were carried out in 30 μL reactions with 15 μL of Phusion high-fidelity PCR Master Mix (New England Biolabs, Ipswich, MA, USA), 0.2 μM of forward and reverse primers, and about 10 ng template DNA. The PCR amplifications were carried out using the following program: 1 min initial denaturation at 98 °C, 30 cycles of 10 s at 98 °C, 30 s at 50 °C, and 30 s at 72 °C, with a final 5 min elongation at 72 °C. Both a negative control (ddH_2_O with no DNA template) and a positive control sample (an artificial DNA molecule with multiple primer sites) were used to assess obvious contamination during sample preparation for PCR and the efficiency of PCR, respectively. The amplicon libraries were sequenced on an Illumina NovaSeq 6000 platform at Novogene Biotech (Beijing, China) to produce 250-bp paired-end reads.

### Metabarcoding data processing and statistical analysis

The sequence quality was checked using FastQC [27]. Raw sequence processing and swarm clustering were performed using the FROGS pipeline (v3.2.3) [28, 29]. The unmerged reads were retained during this process. OTUs with an overall read number of < 200 were discarded, as were those present in fewer than 16 samples (Supplementary Table S1). The rDNA ITS region was identified using ITSx and used for OTU affiliation using the UNITE database (v8.2) [30]. Fungal ecological guilds were assigned to fungal amplicons using the FungalTrait database [31]. The guild affiliation of the most abundant OTUs has been manually curated owing to the potential mis-classification of trophic lifestyle annotated in the FungalTrait database. Normalization based on the lowest number of reads for all samples was performed using random sampling.

To characterize how microbial populations differed across forest associations and soil depths, we used R vegan (v2.5–7) and phyloseq (v1.28.0) packages [32, 33]. The mean species diversity of each sample (alpha diversity) was calculated based on species abundance and observed richness. The mean species diversity between samples (beta diversity) was estimated using the Bray–Curtis method. Data structures were visualized using non-metric multidimensional scaling (NMDS), and variations were quantified using permutational multivariate analysis of variance (PERMANOVA, 9999 permutations). Variations in alpha-diversity according to the soil physicochemical parameters were quantified using the linear model implemented in the R vegan algorithm for constrained ordination [33]. To identify taxonomic and functional differences among tree species and among seasons, Kruskal–Wallis with FDR correction and post-hoc Wilcoxon tests were performed. Differences among the soil layers were analyzed using the Wilcoxon test with FDR correction. These differences were also assessed using DESeq2 implemented in FROGS. As OTU distributions based on rDNA ITS1 and ITS2 were similar, only the results based on rDNA ITS2 are shown. We used the *species affinis* (commonly abbreviated to *aff*.) to indicate that available material or evidence suggests that the proposed species is related to but is not identical to the species with the binomial name until further morphological characterization.

### RNA sequencing of soil eukaryotic mRNA

Approximately two g of sieved soil samples collected during the wet season in August 2019 were extracted using the RNeasy PowerSoil Total RNA kit (QIAGEN, catalog no. 12866–25), according to the manufacturer’s recommendations (for a total of 72 samples; north and southward soil samples were pooled). The final total RNA pellet was suspended in 60 µL RNAse-free water, and its integrity was checked using an Agilent 2100 Bioanalyzer. To eliminate any contaminating DNA, the RNA was incubated with DNase (Promega, RQ1 RNase-Free DNase, catalog no. M6106) at 37 °C for 30 min, incubated at 65 °C for 10 min, immediately placed on ice, and then purified using the One-Step PCR Inhibitor Removal Kit (ZYMO Research, catalog no. D6030), and RNA Clean & Concentrator kit (ZYMO Research, catalog no. R1017). Eukaryotic poly-A RNA was amplified using the MessageAmp II kit, according to the manufacturer’s guidelines (Invitrogen, Thermo Fisher Scientific). RNA integrity and quantity were measured using the RNA Nano 6000 Assay Kit on the Bioanalyzer 2100 system.

The amplified RNA was reverse-transcribed into cDNAs, according to the manufacturer’s protocol (Novogene). cDNAs were then sequenced on an Illumina NovaSeq sequencer using HiSeq TruSeq SBS sequencing kit v4 (250 bp paired-end reads). Adapter sequences were removed from raw reads and low-quality reads were discarded. rRNA was removed using SortMeRNA. The reads were assembled into cDNA contigs using MEGAHIT (v1.1.324) [34]. Six distinct assemblies were produced, corresponding to the organic (OS) and organo-mineral (OM) soil layers for the oak, spruce, and fir forests.

For each of the 72 samples, filtered reads were mapped onto the corresponding de novo metatranscriptome assemblies using Bowtie2 (v2.3.0) [35]. Counts were determined using SAMtools (v1.7) [36]. Contigs supported by less than 10 mapped reads or present in less than four different samples were discarded. These contigs were annotated using Diamond (v2.0.15) [37] and the following parameters (more_sensitive –max-target-seq 1 –max-hsps 1 –evalue 1e^−10^) and two different databases: GenBank non-redundant (April 2021 release) and the Joint Genome Institute MycoCosm (January 2023 release) [38]. Only published genomes were used. Conflicting taxonomic annotations were solved based on a higher Diamond bit score, with an advantage given to MycoCosm in the case of tied scores. Up to 38% of the reads were mapped to the reference fungal genomes. Contigs that were not annotated as fungi were discarded. Read counts were reported as transcripts per million (TPM).

Functional annotations were based on (1) eukaryotic orthologous groups (KOG) annotations available for each genome in the MycoCosm database [38], (2) the CAZy database (April 2023 release) [39], and (3) the MEROPS peptidase database (January 2023 release) [40]. CAZyme annotation was performed on the longest coding sequence (CDS) predicted for each contig. CDS prediction was performed using TransDecoder (v. 5.5.0) (incl. CDS > 100 amino acids) (https://github.com/TransDecoder/). Plant cell wall-degrading enzymes (PCWDE), fungal cell wall-degrading enzymes (FCWDE), and other microbial cell wall-degrading enzymes (neither PCW nor FCW) were annotated according to Miyauchi et al. [21] and Lebreton et al. [41]. Several fungal species found in Xulongxueshan forests are endemic to southwestern China and lack a reference genome. Thus, the RNA reads from their extracted soil mycelium mapped to the available reference genome for closely related species. For example, RNA reads from the ectomycorrhizal *Amanita flavopantherina*, very abundant in Yunnan forest soils, mapped to the reference genomes deposited at JGI MycoCosm of the phylogenetically-related *A. muscaria* from North America, but we considered these RNA reads belong to *A. flavopantherina.* A detailed description of scripts, including parameters and versions used in the workflow, is available at https://github.com/anlebreton/Scripts_from_Zeng_Lebreton_et_al._2023.git.

Ordination plots were generated using the vegan R package v2.5.6. Bar and boxplots were generated using the ggplot2 and ggsignif R packages. Heatmaps were generated using the pheatmap R package and ternary plots were generated using the ggtern package. Differences in expression between fungal guilds were tested using the Wilcoxon test, with FDR-adjusted *p*-values.

## Results

### The soil mycobiome is mainly shaped by forest associations

**Fig. 2:**
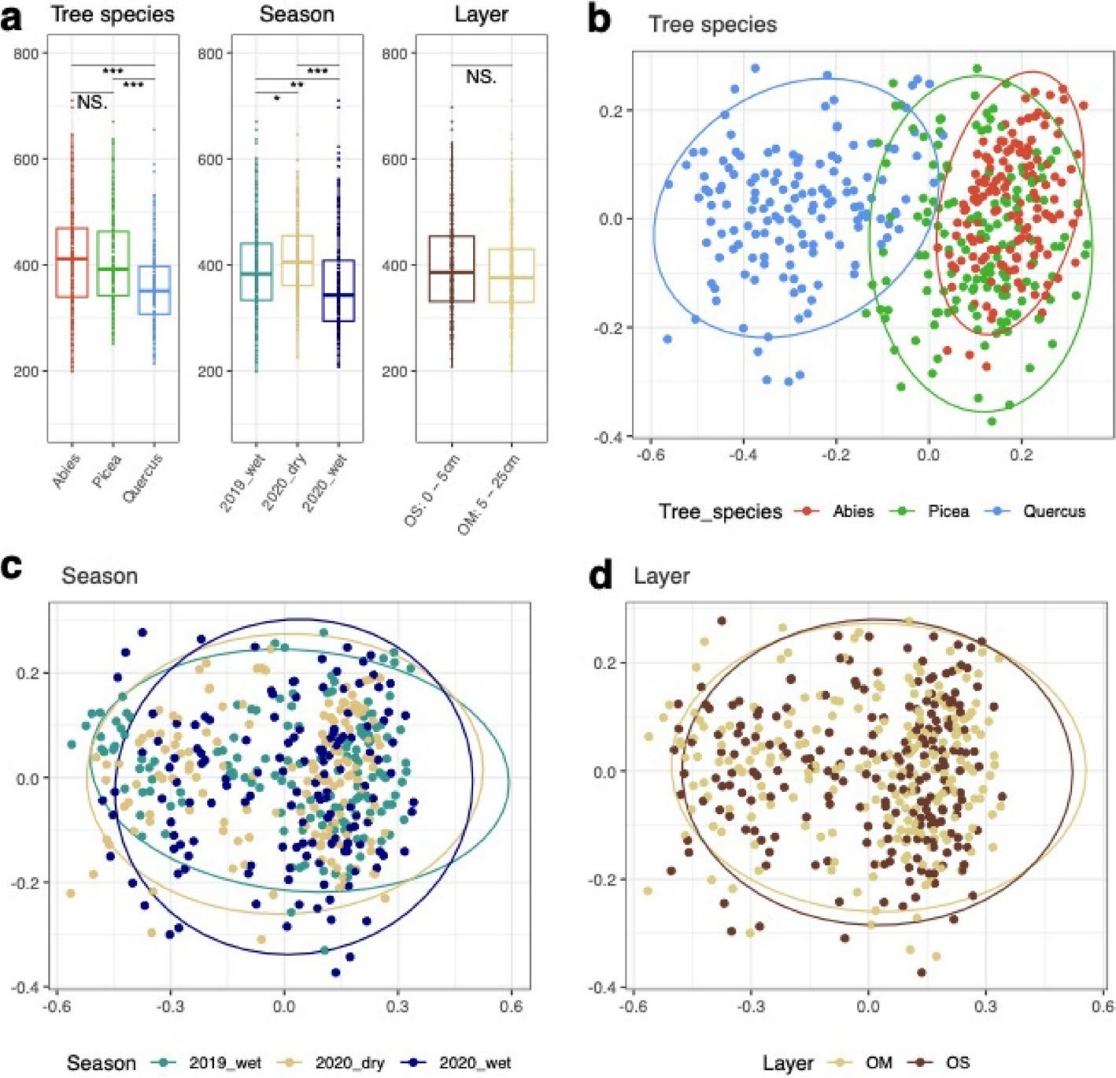
**a** Alpha diversity of fungal communities according to forest associations dominated by fir (*A. forrestii*), spruce (*P. likiangensis*), oak (*Q. pannosa*), season (wet or dry), and soil layer (OS, OM); confidence interval (95%); asterisks indicate significance (*p* ≤ 0.001). **b,c,d** Fungal community dissimilarities between all pairs of sites (*Abies-, Picea-, Quercus*-dominated forests) and between seasons and soil layers using Bray–Curtis dissimilarities based on the relative abundance of OTUs (rDNA ITS2). Data structures were visualized using non-metric multidimensional scaling (NMDS)

In total, we identified 2526 fungal OTUs (based on rDNA ITS2) in the soil cores sampled from the three forest sites (Supplementary Table S1). The observed alpha diversity of the fungal community was highly similar across the two conifer (*P. likiangensis* and *A. forrestii*) forests, and was significantly lower in the oak (*Q. pannosa*) forest (Fig. 2A). This alpha diversity was slightly higher during the dry season than during the rainy monsoon seasons. It was lower in the 2020 wet season compared to 2019 suggesting yearly variations. The alpha diversity was identical in the OS and OM soil layers. We found a significant correlation between the alpha diversity of soil fungi and SOM, N and Ca content, and pH and CEC (Supplementary Fig. S3). In contrast, no correlation was found between alpha diversity and tree growth (as assessed by DBH) or soil P content. NMDS ordination of soil fungal communities illustrated a clear contrast by forest associations, although the conifer communities, that is, spruce- and fir-dominated forests, partially overlapped (Fig. 2BCD). Seasonality and soil layer explained only a minor proportion of the variation in community structure (beta diversity) (Fig. 2CD, Supplementary Table S2).

### The soil mycobiome is dominated by ectomycorrhizal Russula species

**Fig. 3:**
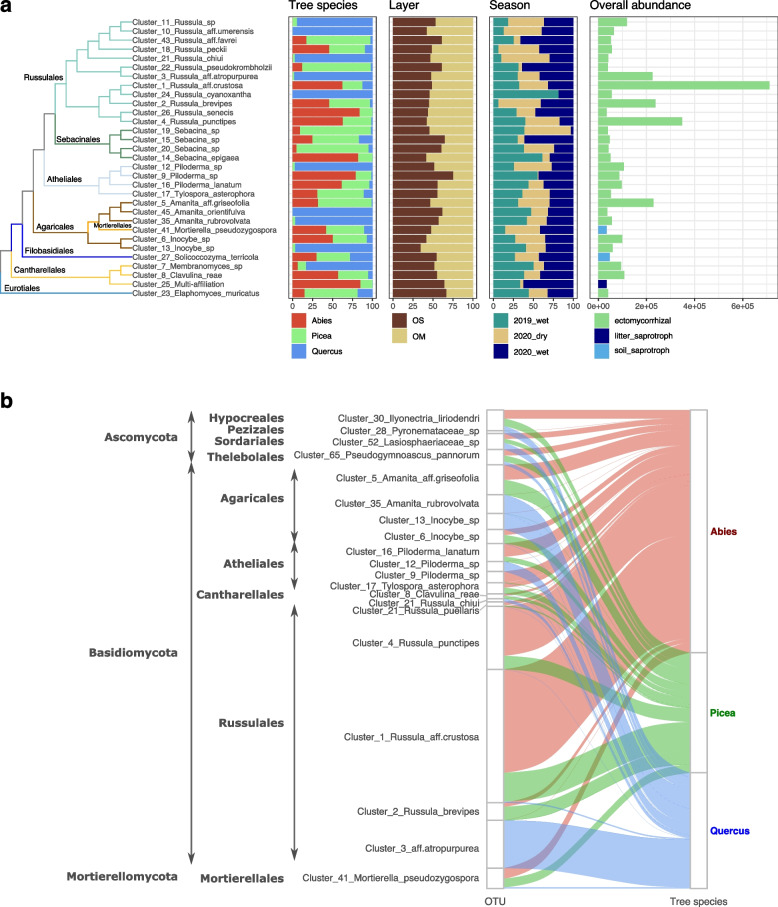
Taxonomic structures of soil fungal communities in oak-, spruce-, and fir-dominated forests. **a** Distribution of the most abundant fungal OTUs (containing > 50% of the total rDNA ITS1 read abundance) according to tree species (*Abies*, *Picea*, *Quercus*), soil layer (OS, OM), and season (wet/dry). The phylogenetic relationships between OTUs are shown on the left side at the order and species levels. The proportion of OTUs (in %) is displayed in the middle panel according to the dominant tree species, the soil layers and the seasons. The overall abundance of the OTUs (in K reads) is shown in the right panel. **b** Network of fungal OTUs associated with each tree species (*Abies, Picea, Quercus*). This shows the frequency of occurrence of the 20 most abundant OTUs displaying significant differences among tree species (Kruskal–Wallis with FDR correction), that is, host preference. The phylogenetic affiliations of OTUs are shown on the left side at the division, order and species levels. *Abies*-associated OTUs are shown in pink, *Picea*-associated OTUs in green and *Quercus*-associated OTUs in blue

Based on rDNA ITS distribution, Basidiomycota dominated the soil fungal community (Fig. 3). OTUs of Glomeromycotina were barely detectable (< 1%). The fungal community primarily consisted of Russulales, Agaricales, and Atheliales, regardless of soil layer or sampling season (Fig. 3A). At the species level, the composition of the mycobiome was highly variable between adjacent broad-leaved (oak) and conifer (spruce or fir) plots (Fig. 3). The core fungal microbiome comprised only ~ 20% of the total OTUs across all soil samples, although the overlap between conifer-associated communities was substantial (Fig. 3A). This taxonomic variability was mainly observed at the species level, and not at higher taxonomic levels (i.e., orders). For example, among the most abundant OTUs found beneath oak, fir, and spruce, ectomycorrhizal *Russula* species exhibited consistent dominance across seasons and soil layers; 50% of the sequences belonged to 31 OTUs only, half of which were assigned to the *Russula* genus (Fig. 3A). Several *Russula* species were specific or showed a strong preference to a tree species, e.g., *R. chui*, *R. cyanoxantha*, *Russula* aff. *umerensis*, and *Russula* aff. *atropurpurea* were only found beneath oaks, while *R. senecis* and *R. brevipes* were only sampled beneath conifers (Fig. 3B). In addition, several species were enriched in the OS or OM layer; *R. favrei*, *R. pseudokrombholzii*, cluster_15_*Sebacina* sp*.* or cluster_9_*Piloderma* sp., were more abundant in the OS than in the OM layer (DESeq2 (LFC > 5; p.adj < 0.05). Several OTUs in *Lactifluus*, *Russula*, *Sebacina*, and *Piloderma* were the most abundant during the rainy monsoon season, while a few taxa within these genera were the most abundant during the dry season (Fig. 3A, Supplementary Fig. S4). The differential spatial and seasonal distributions (Fig. 3A), as well as host preference (Fig. 3B), indicated clear differences in niche/habitat preference, even within functional guilds (saprotrophs vs. ectomycorrhizal) and clades (e.g., *Russula*). This suggests that deterministic niche partitioning in fungal community assembly is a major driver.

### Gene expression profiling of the soil fungal community

**Fig. 4:**
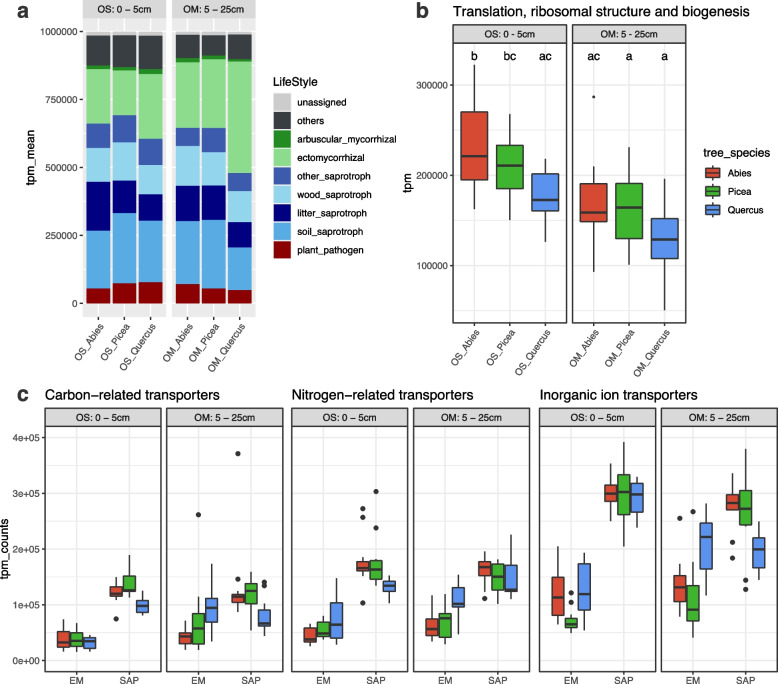
Fungal transcriptional responses to forest associations and soil layers. **a** Relative abundance (in TPM) of RNA transcripts according to the functional fungal guilds. **b** Aggregated sum of transcripts (TPM) mapped to genes assigned to KOG class J fungal transcripts coding for the core translational machinery (KOG class J), a proxy for fungal growth rate. **c** Differences in relative transcription levels of genes involved in the uptake of carbon compounds, nitrogen compounds, and inorganic ions in saprotrophic (SAP) and ectomycorrhizal (EM) fungi. The relative transcription levels of all genes for a specific function (mean ± 1 S.E.) in relation to all protein-coding genes in each sample are shown. Boxplots are Tukey’s test, where the center indicates the median, lower, and upper hinges on the first and third quartiles, respectively, and each whisker is 1.5 × the interquartile range (IQR) from its hinge

To delineate the link between taxonomic composition and functional traits, we examined gene expression patterns of the soil mycobiome using mRNA sequencing. After RNA read assembly, we identified 406,373 unique fungal mRNA contigs (> 500 bp), mostly affiliated with Basidiomycota (mean = 49.9%, min. = 28.7, max. = 77.4), Ascomycota (mean = 22.6%, min. = 9.1, max. = 43.8), Mortierellomycota (mean = 9.5%, min. = 3.3, max. = 33.3), and Mucoromycota (mean = 7.0%; min. = 1.0, max. = 20.6). These transcripts belonged to ectomycorrhizal symbionts (25.2%), soil saprotrophs (22.3%), litter decomposers (12.5%), wood decayers (12.6%), other saprotrophs (8.5%), and plant pathogens (6.3%) (Fig. 4A, Supplementary Fig. S5). The proportion and amounts of transcripts from ectomycorrhizal species were significantly highest in the OM soil layer (5–25 cm) of the oak forest, whereas they were found in similarly lower proportions in the OM/OS layers of both spruce and fir forests, and the OS layer of the oak forest (Fig. 4A, Supplementary Fig. S5). In contrast, transcripts of soil/litter saprotrophs and wood decayers were significantly most abundant in the OM/OS layers of the spruce- and fir-dominated plots (Supplementary Fig. S5).

In the OM layer of the fir forest, the ectomycorrhizal *Tuber zhongdianense*, *R. brevipes* and *A. flavopantherina*, together with the saprotrophic *Basidioascus undulatus, Mortierella* sp*.* and *Linnemannia elongata* were the most transcriptionally active species (Supplementary Table S3). In the OS layer, the mycelium with the highest transcriptional activity belonged to the ectomycorrhizal *Imleria badia* and *Piloderma sphaerosporum*, and the saprotroph *Mycena pura* and *Panus rudis* (Supplementary Table S3). In the spruce OM, the highest activity was observed in the ectomycorrhizal *Phlegmacium (Cortinarius) glaucopus*, *A. flavopantherina*, saprotrophic *Geastrum triplex*, and *Mortierella* sp., whereas in the OS, it was observed in the ectomycorrhizal *Elaphomyces granulatus* and *P. glaucopus*, saprotrophic *Umbelopsis* sp., and *Mortierella* sp. (Supplementary Table S3). In the oak OM layer, the ectomycorrhizal symbionts *A. flavopantherina*, *R. vinacea*, and *Lactarius psammicola* dominated the transcriptome landscape, whereas several ectomycorrhizal *Cortinarius* species were most active in the OS layer (Supplementary Table S3).

The levels of abundance-normalized transcripts encoding proteins of the translation, ribosomal structure, and biogenesis machinery (KOG class J), known to be correlated with rapid growth rate, varied substantially across forest associations (Fig. 4B). These results indicated higher KOG J gene expression (i.e., growth rate) in the near-surface OS soil layer of the three forests than in the deeper OM soil layer (Fig. 4B), with mRNA from saprotrophic taxa dominating the metatranscriptome landscape, except for those beneath oaks. As expected, transcripts related to translation, ribosomal structure and biogenesis (KOG class J), post-translational modification, protein turnover, and chaperones (KOG class O) dominated the transcriptomes of saprotrophic and ectomycorrhizal taxa in the three forests (Supplementary Fig. S6). Some transcripts, such as those belonging to class U (intracellular trafficking, secretion, and vesicular transport), were more abundant in oak-associated fungi. Interestingly, the observed recurring structure of the metatranscriptome contrasted with the large changes observed in the composition of the community at the species level; that is, the metatranscriptome more strongly reflects ecosystem functions rather than its species content.

The hyphal networks of ectomycorrhizal fungi that permeate the soil layers play a key role in the uptake, transport, and assimilation of N compounds, carbohydrates, lipids, and inorganic ions [12]. Therefore, we compared the expression of genes encoding membrane transporters for C and N compounds and inorganic ions in the ectomycorrhizal and saprotrophic taxa (Fig. 4C). Contrary to our expectations, the abundance of transcripts encoding ectomycorrhizal transporters was significantly lower than those of transcripts from saprotrophic fungi (Fig. 4C) in both the OS and OM layers, except for the ectomycorrhizal symbionts of oak trees, which presented a higher level of expression for genes encoding inorganic ion transporters in the OM layer (Fig. 4C). Overall, forest associations had only a very limited impact on the relative transcriptional activity of these fungal genes.

The levels of transcripts for genes related to the transport and metabolism of amino acids, carbohydrates, lipids, and inorganic ions varied strikingly across ectomycorrhizal and saprotrophic OTUs, according to forest associations and soil layers (Supplementary Fig. S7). For example, *A. flavopantherina, R.* aff. *ochroleuca, R.* aff*. vinacea, R.* aff. *seminuda*, and *Wilcoxinia mikolae* accumulated higher levels of transcripts for amino acid transport and metabolism in the OM layer of the oak forest, whereas transcripts from the saprotrophic *Mortierella* sp. and ectomycorrhizal *Trichophaea hybrida* were enriched in the OM layer beneath spruce (Supplementary Fig. S7A). The highest levels of transcripts for carbohydrate transport and metabolism were produced by soil saprotrophic species, such as *Syncephalastrum* aff. *racemosum* and *Cunninghamella* aff*. echinulate* (Mucorales), *Mortierella* aff. *capitata* and *M.* aff. *alpina* (Mortierellales), *Mycena* aff*. pura* (Agaricales) and *Basidiobolus* aff. *meristosporus* (Entomophthorales), although the ectomycorrhizal species *A. flavopantherina*, *R.* aff. *ochroleuca* and *R.* aff. *rugulosa* expressed substantial levels of transcripts beneath oak trees (Supplementary Fig. S7B). Interestingly, species expressing high levels of transcripts for lipid transport and metabolism (Supplementary Fig. S7D) were often ectomycorrhizal species, such as *Thelephora ganbajun* and *Hebeloma* aff*. brunneifolium,* accumulating high levels of these transcripts in the oak OM layer. In contrast, the saprotrophic *M.* aff. *capita, Mycena* aff. *pura*, *Saksenaea* aff. *vasiformis*, *Fennellomyces* sp. presented the highest transcript levels in the spruce OS layer (Supplementary Fig. S7D). Notably, the mycoparasite *Syncephalis* aff*. pseudoplumigaleata* (Zoopaginomycotina) showed the highest transcript levels in the oak OS layer. Regarding the uptake, transport, and assimilation of inorganic ions (Supplementary Fig. S7C), the ectomycorrhizal *Hebeloma* aff. *brunneifolium*, *A. flavopantherina*, and several *Russula* species were highly active in the oak OM layer, whereas the saprotrophic *Chalara* aff. *longipes* and *Haplosporangium* sp. were highly active in the spruce OS layer.

**Fig. 5:**
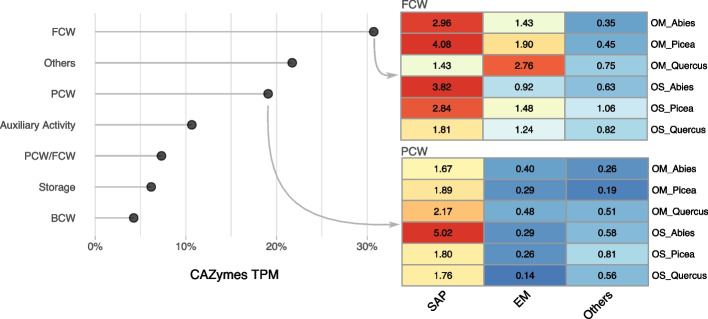
Transcriptional responses of CAZyme genes in soil fungi to tree species and soil layers. **a** The bar graph displays the aggregated sum of transcripts (TPM) mapped to the CAZyme genes expressed in the OS and OM layers of the *Abies-*, *Picea*-, or *Quercus*-dominated forests. Only the most highly transcribed CAZyme gene families are shown. Color codes for gene families are shown on the right panel. **b** Bar plots and heat map depicting the abundance of CAZyme transcripts (TPM) ranked by potential substrates according to tree species (*Abies*, *Picea*, *Quercus*) and soil layers (OM and OS). The bar plots show the proportion (%) of transcripts acting on FCW, PCW, or other substrates. The heat map shows the proportion (%) of transcripts for FCW and PCW CAZyme transcripts. Values are in TPM and the color codes range from red (higher expression) to light blue (lower expression). Abbreviations: BCW, bacterial cell walls; FCW, fungal cell walls; PCW, plant cell walls. ‘Auxilliary Activity’ corresponds to CAZyme genes involved in lignin and polyphenol degradation; ‘Storage’ corresponds to CAZyme genes related to the biosynthesis of storage compounds, such as glycogen

The CAZyme transcripts comprised approximately 2% of the total transcriptome. Although the 14 most highly expressed CAZyme gene families involved in polysaccharide and lignin (i.e., SOM) modification and decomposition showed a similar profile in the soil layers of the three forest plots, we observed varying proportions of glycosyl hydrolase (GH), carbohydrate esterase (CE), glycosyl transferase (GT), and auxiliary enzyme (AA) transcripts (Fig. 5A, Supplementary Table S4). The gene expression of CAZymes acting on different substrates (e.g., plant or fungal cell walls) varied widely among fungal guilds (saprotrophic vs. ectomycorrhizal species) (Fig. 5B). Laccases (AA1), class II peroxidases (AA2), and cellobiose dehydrogenases (AA3), which act on lignin and polyphenols, were among the most highly expressed CAZyme genes (Fig. 5A). Endoglucanases (GH5_9 and GH16_1), ß-glucanases (GH17), β-1,3-glucanase (GH128) and chitinases/endo-β-N-acetylglucosaminidases (GH18) were also highly expressed. Notably, transcripts coding for proteins with LysM domains (CBM50) that bind to N-acetylglucosamine were prominent.

As expected, the expression of CAZyme genes acting on fungal and plant cell wall polysaccharides from saprotrophic taxa dominated the transcript profiles (Fig. 5B). The highest levels were found beneath conifers. The largest proportion of CAZyme transcripts from ectomycorrhizal fungi was found in the OM layer of the oak and spruce forests and they correspond to CAZymes acting on fungal cell wall synthesis/degradation, such as chitin synthase and chitinase (Fig. 5B). Transcripts related to fungal cell walls were also highly expressed by saprotrophic fungi (Fig. 5B) in the three forest plots.

**Fig. 6:**
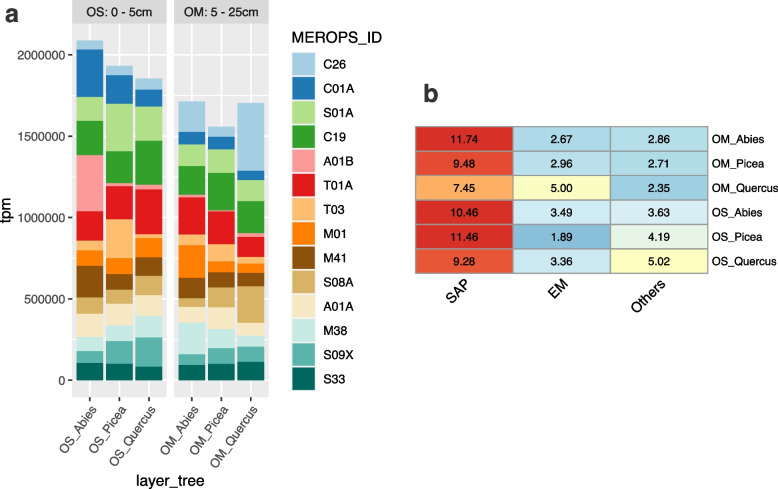
Transcriptional responses of protease genes from soil fungi to tree species and soil layers. **a** The bar graph displays the aggregated sum of transcripts (TPM) mapped to genes assigned to the MEROPS protease families expressed in the OS and OM layers of the *Abies-*, *Picea*-, or *Quercus*-dominated forests. Only the most highly transcribed CAZyme gene families are shown. Color codes for gene families are shown on the right panel. **b** The heat map displays the proportion (%) of the aggregated sum of transcripts (TPM) mapped to the MEROPS protease families for the ectomycorrhizal (ECM) and saprotrophic (SAP) species. The color codes range from red (higher expression) to light blue (lower expression)

The number of transcripts coding for peptidases/proteases accounted for approximately 5% of the total fungal metatranscriptome, with several secreted proteases, such as cysteine proteases (e.g., papain C01), metalloproteases (M01, M41), and serine proteases (S01, S08, S9), among the most abundant transcripts, suggesting a high capacity for protein degradation (Fig. 6A). The distribution of transcripts for the main protease families was very similar in the OS and OM layers of the three forest plots (Fig. 6A). The OM layer of the oak forest was characterized by higher transcription levels of several protease genes from ectomycorrhizal fungi (Fig. 6B), whereas protease genes of saprotrophic species had a much higher level of expression in both the OS and OM layers in the three forests (Fig. 6C).

## Discussion

The main goal of the present study was to gain a better understanding of the impact of forest associations on the assembly and functioning of soil fungal communities to provide mechanistic explanations for the microbially mediated exploitation of soil resources in old-growth forests. Most importantly, profiling of the soil fungal metatranscriptome allowed us to assess the expression of genes related to growth and metabolism, including SOM decomposition and N acquisition, with limited confounding effects from climatic variation or other large-scale environmental drivers.

Soil and litter decomposers, wood decayers, and ectomycorrhizal symbionts accounted for over 80% of the OTUs, whereas other functional guilds, such as arbuscular mycorrhizal symbionts, plant pathogenic fungi, parasites, and yeasts, showed much lower abundance and greater variability. Previous surveys have also found that ectomycorrhizal fungi are abundant in organic soil layers, where they are capable of using nutrients from organic resources leached from aboveground litter [42, 43]. Among the ectomycorrhizal fungi, *Russula* taxa were the most prominent species associated with oak, spruce, and firs. Based on their sheer abundance and wide distribution in old-growth forests and plantations in subtropical Southeast Asia, *Russula* species presumably have great ecological importance as ectomycorrhizal symbionts in old-growth forests [14, 44–46]. The prominence of *Russula* species, characterized by short-distance or contact extramatrical mycelial exploration types, may be related to a fast turnover of decomposing SOM, leading to high pools of labile N [47]. These ectomycorrhizal species expressed among the highest levels of genes related to the transport and metabolism of amino acids, carbohydrates, lipids, and inorganic ions, confirming their prominent functional roles in old-growth montane forests.

As shown in several temperate forest ecosystems [13, 48–50], the forest associations (that is the dominant tree species) was the main factor driving Xulongxueshan fungal communities. Notably, soils of the spruce and fir forests shared more OTUs than oak forest soils, suggesting resource similarities in conifer forest soils that can be exploited by the same species. Additional variables, such as seasonality and soil physicochemical characteristics, explained a minor portion of the variation in alpha and beta diversities in the fungal community composition. Hence, the segregation patterns detected in these montane forests likely reflect mutual exclusion between fungal OTUs that were potentially caused by biotic interactions (e.g., host specificity/preference) or environmental filtering (tree-related SOM composition and/or structure) rather than spatially neutral assembly. Because the most abundant fungal OTUs are phylogenetically related (e.g., *Russula*), environmental filtering appears to act similarly on closely related clades.

Fragments of decaying organic matter and mycorrhizal roots were discarded during soil sample sieving. Therefore, sequenced fungal mRNAs mainly originate from active mycelia proliferating in organic and mineral soil layers (e.g., mycorrhizal networks), although minor amounts of mRNAs can also be stored in dormant sexual/asexual spores, stromata, and other resting structures. Our gene expression profiling therefore missed one of the most active parts of the mycorrhizal networks, the ectomycorrhizal roots. This likely explains the lower proportion of transcripts from ectomycorrhizal fungi compared to saprotrophic species in the total soil metatranscriptome, while ectomycorrhizal symbionts appeared to be the most abundant fungi based on the rDNA ITS-based surveys. The latter molecular marker likely missed considerable portions of the active fungal OTUs, as previously reported by Schneider et al. [51] in Norway spruce ectomycorrhizal roots. In addition, soil fungal DNA may survive for long periods after hyphal death, potentially confounding efforts to study living, transcriptionally active mycelia.

We expected that the observed striking differences in mycobiome composition (at the OTU level) would be mirrored in the major cellular and molecular functions expressed in soils beneath oak, fire, and spruce trees. We found differences in the total transcriptional activity of the fungal community according to the dominant tree species and the soil layer. The highest transcriptional activity (for KOG class J genes, a proxy for growth-rate) was measured in the spruce OS layer, whereas the lowest was found in the oak OM layer. On the other hand, the RNA-seq profiles revealed that the cellular and metabolic functional structure of fungal communities, in terms of the relative abundance of transcripts coding for the core functional gene categories (e.g., KOG J, O, nutrient transporters), were similar among all forest associations and within each fungal guild. Although the overall distribution patterns for transcripts coding CAZyme and MEROPS gene families was similar, we observed substantial differential quantitative gene expression for these pathways related to polysaccharide and protein degradation. This functional similarity of core metabolic and growth-related pathways is presumably promoted by a strong stoichiometric balance between coupled cellular and metabolic pathways, the majority of which sustain the growth rate. Although genes related to polysaccharide and protein degradation were expressed at a significant rate (see below), both saprotrophic and ectomycorrhizal fungi preferentially invested in mechanisms that would ensure their growth and survival, such as cell metabolic maintenance. A substantial number of extracellular proteases, together with oligopeptides and amino acid transporters, are likely indicative of N scavenging within the N-limited substrate. Here, we showed that genes related to inorganic ion, carbohydrate, and amino acid transport and metabolism are highly expressed in several saprotrophic and ectomycorrhizal fungi, supporting the key role of mycelial networks in nutrient scavenging and assimilation to support soil and tree metabolism.

We confirmed that genes related to degrading enzymes involved in the decomposition of soil plant, fungal, and bacterial cell wall polysaccharides were distributed across multiple fungal decomposers [10] and major functional guilds (i.e., soil saprotrophs and ectomycorrhizal fungi). The decomposition of plant, fungal, and bacterial polysaccharides involved specific fungal clades that vary across forest types. Genes encoding enzymes acting on plant and fungal cell wall polysaccharides dominated the CAZyme RNA profiles of the OS and OM layers. As expected, saprotrophic taxa expressed higher levels of multicopper oxidases (AA1), class II lignin-modifying peroxidases (AA2), oxidoreductases (AA3), glucanases (GH16 and GH128), chitinase (GH18), and associated chitin-binding modules (CBM50), confirming the expression of a diverse array of degrading enzymes acting on plant and microbial polymers. The frequent detection of GT2 type sequences in our data indicates the presence and expression of genes encoding periplasmic integral membrane chitin synthases, confirming that fungi are metabolically active in various soil layers.

In each forest association and soil layer, the same metabolic niches appear to be occupied by different fungal taxa, often belonging to the same genus (for example, *Russula*), even if the occupancy of each niche in terms of its relative abundance by a guild (symbiotrophic *vs.* saprotrophic) remains almost unchanged. Our study demonstrates that taxonomic variability between forest ecosystems does not imply major differences in community functions, such as nutrient uptake and SOM decomposition. The striking taxonomic variability within each functional guild is presumably enabled by high functional redundancy in the regional fungal pool, allowing for potential colonization of each soil and dominant tree by alternative, functionally similar OTUs. The precise mechanisms determining the subset of OTUs that eventually establish in oak-, fir- or spruce-dominated ecosystems and within each metabolic niche/soil layers are, at this point, unknown, but likely driven by host preference. Our findings point to an important difference between functional and taxonomic structures in fungal communities, which may arise because mechanisms leading to a convergence of cellular and metabolic functions do not necessarily lead to convergence of taxonomic composition. Reciprocally, strong taxonomic turnover may only weakly affect ecosystem functioning, as the gene repertoire of fungal species within a genus, for example *Russula*, is often very similar even if idiosyncrasies can be noted between taxa [41, 52]. In our study, heterogeneous environments related to variations in several physicochemical features, such as pH, N and P content, and SOM composition, may have driven taxonomic diversity without affecting the main expressed functions.

In conclusion, this study provides valuable insights into the complex relationships between forest types, fungal communities, and soil characteristics. By linking genes to ecological traits, our findings underscore the need for an efficient assessment of the diversity and distribution of functional genes and their producers, which can now be achieved through soil RNA sequencing. Moreover, our study suggests that gene expression patterns offer a more reliable indicator of ecosystem functioning than functional traits, based only on taxonomic composition. Future research should aim to elucidate the intricate signaling and metabolic pathways involved in plant–microbe and microbe-microbe interactions to inform biogeochemical models describing old-growth forests. Ultimately, this knowledge can help to predict the impact of environmental and anthropogenic activities on microbial biodiversity and facilitate the development of effective conservation strategies of threatened ecosystems.

### Supplementary Information

**Additional file 1: Supplementary Table S1.** Filtering steps of rDNA ITS1 and ITS2 reads and their impact on the number of fungal OTUs. **Supplementary Table S2.** PERMANOVA on the observed alpha diversity of the fungal community based on rDNA ITS2 primers. **Supplementary Table S3.** Transcriptional activity of the 10 most transcriptionally active fungal species in the OS and OM soil layers of fir, spruce, and oak forests. **Supplementary Table S4. **The most abundant cDNA contigs encoding CAZymes assembled from RNA reads from the OS and OM soil layers of fir, spruce, and oak forests. **Supplementary Figure S1.** Annual precipitation and temperature at the Lijiang Forest Biodiversity National Observation and Research Station in Yunshanping, Yulongxueshan, NW Yunnan Province (WorldClim database, https://www.worldclim.org). Arrows indicate the sampling periods during the dry and wet (monsoon) seasons. **Supplementary Figure S2.** Physicochemical properties of OS and OM soil layers in fir-, spruce-, and oak-dominated forest stands. NS. not significant, * *p* < 0.05, ** *p* < 0.01, *** *p* < 0.001 using the Wilcoxon test. **Supplementary Figure S3.** Correlation between alpha diversity of the soil fungal community, major soil physicochemical characteristics, and tree productivity across the three forest sites. The measured soil features were soil organic matter (SOM), nitrogen (N), Corg/N ratio, pH, calcium, cationic exchange capacity (CEC), phosphorus (P), and tree productivity (diameter at breast height [DBH]). The solid lines indicate significant regression lines. The levels of significance and coefficients of determination (R^2^) for all lines are shown in plots with a confidence interval (95%). **Supplementary Figure S4.** Distribution of fungal OTUs in soil cores sampled during the dry or wet seasons. The abundance of OTUs (based on rDNA ITS2) was displayed on a logarithmic scale. OTUs with significant differential abundance were detected using pairwise comparisons with DESeq2 (|LFC| > 5; *p*.adj < 0.05), followed by forest plots (oak, spruce, and fir) and seasons (dry and wet). Clustering was performed using Ward’s D2 method. Tree species, seasons, fungal phylum and orders, and functional guilds are color-coded as depicted in the right panel. The relative abundance of OTUs is represented by a color scale from the lowest (white) to the highest (dark blue) levels. **Supplementary Figure S5.** Fungal transcriptional responses to forest associations and soil layers. Relative abundance (in TPM) of RNA transcripts according to the functional fungal guilds, i.e., ectomycorrhizal fungi (EM), saprotrophic fungi (SAP), plant pathogens and others in the OS and OM layers of the *Abies*-, *Picea*-, or *Quercus*-dominated forests. Green, blue, black and red box plots indicate the abundance of transcripts for ectomycorrhizal, saprotrophic and pathogenic fungi, and other guilds, respectively. Letters represent the results of a TukeyHSD post hoc test of an ANOVA model with a confidence level of 0.95. **Supplementary Figure S6.** Transcriptional responses of genes related to core developmental, cellular, and metabolic pathways in tree species and soil layers. Distribution of soil fungal transcripts among KOG groups (a) and classes (b) according to forest associations (oak, spruce, and fir) and soil layers (OM and OS). The bar graphs depict the aggregated sum of transcripts mapped to the genes assigned to the different KOG groups and classes for different fungal lifestyles. **Supplementary Figure S7.** Transcriptional responses of nutrition-related genes to tree species and soil layers. Heat maps depict an aggregated sum of transcripts mapped to the genes assigned to carbohydrate transporters and assimilation enzymes (a), amino acid transporters and assimilation enzymes (b), inorganic ion-related transporters and enzymes (c), and lipid-related transporters and enzymes (d) in soil fungi according to tree species (oak, spruce, and fir) and soil layer (OM, OS). Lifestyles, soil layers, and tree species are color-coded, as indicated in the right panel. The data were clustered using complete linkages, according to similar abundance patterns. The abundance of transcripts (in log scale) is represented by a color scale from the lowest (white) to the highest (black) levels.

## Data Availability

Raw sequencing data were deposited in the NCBI Sequence Read Archive database under the accession numbers PRJNA957854 (for rDNA ITS1), PRJNA957896 (for rDNA ITS2), and PRJNA955425 (for RNA reads). The datasets supporting the conclusions of this article are included within the article and its additional files (Supplementary Material). Scripts used for data analysis are available at GitHub: https://github.com/anlebreton/Scripts_from_Zeng_Lebreton_et_al._2023.git
