# Supplementary material for: Stable functional structure despite high taxonomic variability across fungal communities in soils of old-growth montane forests
Source: Microbiome. 2023 Oct 2;11:217. doi: 10.1186/s40168-023-01650-7 (PMC10544587; doi:10.1186/s40168-023-01650-7)
Supplement: Supplementary file 2 — Additional file 1: Supplementary Table S1. Filtering steps of rDNA ITS1 and ITS2 reads and their impact on the number of fungal OTUs. Supplementary Table S2. PERMANOVA on the observed alpha diversity of the fungal community based on rDNA ITS2 primers. Supplementary Table S3. Transcriptional activity of the 10 most transcriptionally active fungal species in the OS and OM soil layers of fir, spruce, and oak forests. Supplementary Table S4. The most abundant cDNA contigs encoding CAZymes assembled from RNA reads from the OS and OM soil layers of fir, spruce, and oak forests. Supplementary Figure S1. Annual precipitation and temperature at the Lijiang Forest Biodiversity National Observation and Research Station in Yunshanping, Yulongxueshan, NW Yunnan Province (WorldClim database, https://www.worldclim.org). Arrows indicate the sampling periods during the dry and wet (monsoon) seasons. Supplementary Figure S2. Physicochemical properties of OS and OM soil layers in fir-, spruce-, and oak-dominated forest stands. NS. not significant, * p < 0.05, ** p < 0.01, *** p < 0.001 using the Wilcoxon test. Supplementary Figure S3. Correlation between alpha diversity of the soil fungal community, major soil physicochemical characteristics, and tree productivity across the three forest sites. The measured soil features were soil organic matter (SOM), nitrogen (N), Corg/N ratio, pH, calcium, cationic exchange capacity (CEC), phosphorus (P), and tree productivity (diameter at breast height [DBH]). The solid lines indicate significant regression lines. The levels of significance and coefficients of determination (R2) for all lines are shown in plots with a confidence interval (95%). Supplementary Figure S4. Distribution of fungal OTUs in soil cores sampled during the dry or wet seasons. The abundance of OTUs (based on rDNA ITS2) was displayed on a logarithmic scale. OTUs with significant differential abundance were detected using pairwise comparisons with DESeq2 (|LFC| > 5; p.adj < 0.0 [file 40168_2023_1650_MOESM1_ESM.docx]

**Supplementary Material**

Stable functional structure despite high taxonomic variability across fungal communities in soils of old-growth montane forests

by Qingchao Zeng, Annie Lebreton, Lucas Auer, Xiaowu Man, Liukun Jia, Gengshen Wang, Sai Gong, Vincent Lombard, Marc Buée, Gang Wu, Yucheng Dai, Zhuliang Yang and Francis M. Martin

*******

**Supplementary Table 1.** Filtering steps of rDNA ITS1 and ITS2 reads and their impact on the number of fungal OTUs.

| **Steps** |  |  | **ITS1** | **ITS2** |
| --- | --- | --- | --- | --- |
| Sequencing output | Raw reads per file | min | 22,409 | 17,980 |
|  |  | max | 99,936 | 82,802 |
|  | Number of sequences |  | 35,737,984 | 29,822,715 |
| Merging process | Reads per file after merging | min | 19,945 | 16,408 |
|  |  | max | 95,853 | 79,079 |
|  | Nbr of sequences after merging |  | 33,271,846 | 27,808,323 |
|  | Nbr of sequences combined (no overlap between R1 and R2) |  | 2,291,139 | 1,964,938 |
| Clustering swarm | Number of clusters |  | 2,541,273 | 2,480,614 |
| Chimera removal | OTUs after chimera removal | Nbr OTUs | 2,317,182 | 2,244,046 |
|  |  | Nbr seq | 32,817,272 | 29,313,125 |
|  |  | % total sequences | 98.6% | 98.5% |
| Filtering | OTUs after filtering - prevalence 16, min reads per OTU = 200 | Nbr OTUs | 2410 | 2708 |
|  |  | Nbr seq | 29,802,905 | 26,163,300 |
|  |  | % total sequences | 90.8% | 89.3% |
| ITSx | OTUs after ITS selection | Nbr OTUs | 2,409 | 2,670 |
|  |  | Nbr sequences | 29802556 | 26092420 |
|  |  | % total sequences | 100.0% | 99.7% |
| Taxonomic affiliation | OTUs with affiliation | Nbr OTUs | 2,401 | 2,627 |
|  |  | Nbr sequences | 29,793,127 | 26,045,170 |
|  |  | % total sequences | 100.0% | 99.8% |
| Removal of multi-affiliation at phylum scale; "no data" and "unidentified" |  | Nbr OTUs | 2,268 | 2,526 |
|  |  | Nbr sequences | 29,417,927 | 25,771,981 |
|  | Nbr conserved reads per file | min | 11,507 | 15,488 |
|  |  | max | 90,189 | 74,373 |
|  | Nbr OTUs per file | min | 247 | 239 |
|  |  | max | 751 | 980 |
| After normalisation | Nbr OTUs per file | min | 121 | 199 |
|  |  | max | 517 | 710 |
|  |  | Nbr OTUs | 2,268 | 2,526 |
|  |  | Nbr sequences | 4,936,503 | 6,675,328 |
|  |  | % remaining sequences compared to input sequences | 13.8% | 22.4% |

| **Supplementary Table 2.** PERMANOVA on the observed alpha diversity of the fungal community based on rDNA ITS2 primers | | | |
| --- | --- | --- | --- |
| **Features** | **R^2^** | **Pr(>F)** |  |
| Tree_species | 0.07104 | 0.0001 | *** |
| Season | 0.05125 | 0.0001 | *** |
| Layer | 0.00196 | 0.3037 |  |
| Tree_species:Season | 0.06061 | 0.0001 | *** |
| Tree_species:Layer | 0.00077 | 0.8541 |  |
| Season:Layer | 0.02495 | 0.0019 | ** |
| Tree_species:Season:Layer | 0.00347 | 0.7835 |  |

**Supplementary Table 3.** Transcriptional activity of the 10 most transcriptionally active fungal species in the OS and OM soil layers of fir, spruce, and oak forests.

| ***PD_Genus*** | ***PD_Species*** | **PD_Family** | **PD_Order** | **LifeStyle** | **OM_Abies** | **OM_Picea** | **OM_Quercus** | **OS_Abies** | **OS_Picea** | **OS_Quercus** |
| --- | --- | --- | --- | --- | --- | --- | --- | --- | --- | --- |
| *Tuber* | *zhongdianense* | Tuberaceae | Pezizales | ectomycorrhizal | 475939 | 378 | 15075 | 56382 | 2731 | 2561 |
| *Basidioascus* | *undulatus* | Geminibasidiaceae | Geminibasidiales | soil_saprotroph | 265550 | 921 | 2467 | 2001 | 4456 | 4606 |
| *Mortierella* | *sp* | Mortierellaceae | Mortierellales | soil_saprotroph | 196030 | 387528 | 116079 | 114638 | 188862 | 100380 |
| *Botryosphaeria* | *dothidea* | Botryosphaeriaceae | Botryosphaeriales | plant_pathogen | 195163 | 1554 | 2249 | 2232 | 2110 | 6619 |
| *Russula* | *brevipes* | Russulaceae | Russulales | ectomycorrhizal | 180896 | 150482 | 31815 | 54494 | 91050 | 7408 |
| *Linnemannia* | *elongata* | Mortierellaceae | Mortierellales | soil_saprotroph | 173263 | 79221 | 26063 | 137975 | 64537 | 31327 |
| *Gymnopus* | *earleae* | Omphalotaceae | Agaricales | litter_saprotroph | 172337 | 1205 | 10324 | 10223 | 3403 | 11127 |
| *Mycena* | *floridula* | Mycenaceae | Agaricales | litter_saprotroph | 154083 | 33020 | 5311 | 29899 | 14865 | 2596 |
| *Cladochytrium* | *replicatum* | Cladochytriaceae | Cladochytriales | litter_saprotroph | 147393 | 29520 | 42785 | 68698 | 40295 | 52300 |
| *Amanita* | *flavopantherina* | Amanitaceae | Agaricales | ectomycorrhizal | 143082 | 359990 | 470893 | 40403 | 96416 | 140416 |

| ***PD_Genus*** | ***PD_Species*** | **PD_Family** | **PD_Order** | **LifeStyle** | **OM_Abies** | **OM_Picea** | **OM_Quercus** | **OS_Abies** | **OS_Picea** | **OS_Quercus** |
| --- | --- | --- | --- | --- | --- | --- | --- | --- | --- | --- |
| *Phlegmacium* | *glaucopus* | Cortinariaceae | Agaricales | ectomycorrhizal | 30813 | 743574 | 15550 | 63996 | 168632 | 50634 |
| *Geastrum* | *triplex* | Geastraceae | Geastrales | litter_saprotroph | 2367 | 441627 | 4497 | 4534 | 6240 | 2239 |
| *Mortierella* | *sp* | Mortierellaceae | Mortierellales | soil_saprotroph | 196030 | 387528 | 116079 | 114638 | 188862 | 100380 |
| *Amanita* | *flavopantherina* | Amanitaceae | Agaricales | ectomycorrhizal | 143082 | 359990 | 470893 | 40403 | 96416 | 140416 |
| *Aspergillus* | *heteromorphus* | Aspergillaceae | Eurotiales | soil_saprotroph | 1734 | 333307 | 11167 | 3948 | 4382 | 6054 |
| *Mortierella* | *alpina* | Mortierellaceae | Mortierellales | soil_saprotroph | 46547 | 151522 | 14239 | 82947 | 73191 | 30671 |
| *Russula* | *brevipes* | Russulaceae | Russulales | ectomycorrhizal | 180896 | 150482 | 31815 | 54494 | 91050 | 7408 |
| *Piloderma* | *sphaerosporum* | Atheliaceae | Atheliales | ectomycorrhizal | 73053 | 143737 | 21965 | 162496 | 128160 | 21010 |
| *Lobosporangium* | *transversale* | Mortierellaceae | Mortierellales | soil_saprotroph | 17998 | 129185 | 9422 | 20468 | 16842 | 12151 |
| *Mariannaea* | *sp* | Nectriaceae | Hypocreales | wood_saprotroph | 737 | 123461 | 362 | 773 | 65204 | 2590 |

| ***PD_Genus*** | ***PD_Species*** | **PD_Family** | **PD_Order** | **LifeStyle** | **OM_Abies** | **OM_Picea** | **OM_Quercus** | **OS_Abies** | **OS_Picea** | **OS_Quercus** |
| --- | --- | --- | --- | --- | --- | --- | --- | --- | --- | --- |
| *Amanita* | *flavopantherina* | Amanitaceae | Agaricales | ectomycorrhizal | 143082 | 359990 | 470893 | 40403 | 96416 | 140416 |
| *Russula* | *vinacea* | Russulaceae | Russulales | ectomycorrhizal | 25921 | 29333 | 403416 | 14003 | 8755 | 118418 |
| *Lactarius* | *psammicola* | Russulaceae | Russulales | ectomycorrhizal | 27798 | 7293 | 393349 | 11228 | 4030 | 58999 |
| *Hebeloma* | *brunneifolium* | Hymenogastraceae | Agaricales | ectomycorrhizal | 39496 | 20512 | 302224 | 2957 | 14578 | 34213 |
| *Mycena* | *haematopus* | Mycenaceae | Agaricales | litter_saprotroph | 2535 | 1060 | 300211 | 9088 | 9006 | 6577 |
| *Lactarius* | *subdulcis* | Russulaceae | Russulales | ectomycorrhizal | 26441 | 10051 | 266524 | 46169 | 2756 | 38775 |
| *Russula* | *ochroleuca* | Russulaceae | Russulales | ectomycorrhizal | 57062 | 47235 | 262707 | 30932 | 14373 | 113288 |
| *Russula* | *rugulosa* | Russulaceae | Russulales | ectomycorrhizal | 48641 | 66521 | 216019 | 33475 | 52224 | 124852 |
| *Russula* | *emetica* | Russulaceae | Russulales | ectomycorrhizal | 51392 | 77019 | 201284 | 43549 | 113035 | 100630 |
| *Chalciporus* | *piperatus* | Boletales | Boletales | saprotroph | 1167 | 944 | 196142 | 2161 | 2931 | 31928 |

| ***PD_Genus*** | ***PD_Species*** | **PD_Family** | **PD_Order** | **LifeStyle** | **OM_Abies** | **OM_Picea** | **OM_Quercus** | **OS_Abies** | **OS_Picea** | **OS_Quercus** |
| --- | --- | --- | --- | --- | --- | --- | --- | --- | --- | --- |
| *Xerocomus* | *badius* | Boletaceae | Boletales | ectomycorrhizal | 54178 | 13905 | 65721 | 470188 | 12012 | 37538 |
| *Mycena* | *pura* | Mycenaceae | Agaricales | litter_saprotroph | 111259 | 2201 | 4351 | 449960 | 88367 | 13309 |
| *Psilocybe* | *serbica* | Hymenogastraceae | Agaricales | litter_saprotroph | 26321 | 12672 | 12729 | 189890 | 2518 | 4620 |
| *Panus* | *rudis* | Panaceae | Polyporales | wood_saprotroph | 6785 | 2371 | 1278 | 170360 | 4824 | 2761 |
| *Piloderma* | *sphaerosporum* | Atheliaceae | Atheliales | ectomycorrhizal | 73053 | 143737 | 21965 | 162496 | 128160 | 21010 |
| *Basidiobolus* | *meristosporus* | Basidiobolaceae | Basidiobolales | other_saprotroph | 30820 | 39630 | 33876 | 145558 | 39693 | 46913 |
| *Linnemannia* | *elongata* | Mortierellaceae | Mortierellales | soil_saprotroph | 173263 | 79221 | 26063 | 137975 | 64537 | 31327 |
| *Serendipita* | *sp* | Serendipitaceae | Sebacinales | endophyte | 76882 | 25298 | 47390 | 136549 | 22400 | 28033 |
| *Mycena* | *sanguinolenta* | Mycenaceae | Agaricales | litter_saprotroph | 5172 | 2609 | 8320 | 115428 | 3366 | 3913 |
| *Mortierella* | *sp* | Mortierellaceae | Mortierellales | soil_saprotroph | 196030 | 387528 | 116079 | 114638 | 188862 | 100380 |

| ***PD_Genus*** | ***PD_Species*** | **PD_Family** | **PD_Order** | **LifeStyle** | **OM_Abies** | **OM_Picea** | **OM_Quercus** | **OS_Abies** | **OS_Picea** | **OS_Quercus** |
| --- | --- | --- | --- | --- | --- | --- | --- | --- | --- | --- |
| *Umbelopsis* | *sp* | Umbelopsidaceae | Umbelopsidales | soil_saprotroph | 43207 | 11336 | 21473 | 102974 | 420844 | 45272 |
| *Elaphomyces* | *granulatus* | Elaphomycetaceae | Eurotiales | ectomycorrhizal | 2271 | 8618 | 12974 | 27809 | 220203 | 32350 |
| *Mortierella* | *sp* | Mortierellaceae | Mortierellales | soil_saprotroph | 196030 | 387528 | 116079 | 114638 | 188862 | 100380 |
| *Phlegmacium* | *glaucopus* | Cortinariaceae | Agaricales | ectomycorrhizal | 30813 | 743574 | 15550 | 63996 | 168632 | 50634 |
| *Coccomyces* | *strobi* | Rhytismataceae | Rhytismatales | litter_saprotroph | 14534 | 435 | 1378 | 1218 | 160558 | 6697 |
| *Plectania* | *melastoma* | Sarcosomataceae | Pezizales | litter_saprotroph | 1580 | 1889 | 2660 | 12649 | 147386 | 2028 |
| *Bjerkandera* | *adusta* | Phanerochaetaceae | Polyporales | wood_saprotroph | 1640 | 6929 | 2222 | 1107 | 139585 | 5896 |
| *Piloderma* | *sphaerosporum* | Atheliaceae | Atheliales | ectomycorrhizal | 73053 | 143737 | 21965 | 162496 | 128160 | 21010 |
| *Fimicolochytrium* | *jonesii* | Powellomycetaceae | Spizellomycetales | other_saprotroph | 17009 | 9413 | 8401 | 21907 | 115184 | 12666 |
| *Russula* | *emetica* | Russulaceae | Russulales | ectomycorrhizal | 51392 | 77019 | 201284 | 43549 | 113035 | 100630 |

| ***PD_Genus*** | ***PD_Species*** | **PD_Family** | **PD_Order** | **LifeStyle** | **OM_Abies** | **OM_Picea** | **OM_Quercus** | **OS_Abies** | **OS_Picea** | **OS_Quercus** |
| --- | --- | --- | --- | --- | --- | --- | --- | --- | --- | --- |
| *Cortinarius* | *saniosus* | Cortinariaceae | Agaricales | ectomycorrhizal | 61836 | 60120 | 38732 | 76629 | 18362 | 188739 |
| *Cortinarius* | *sp* | Cortinariaceae | Agaricales | ectomycorrhizal | 10610 | 12368 | 24798 | 1023 | 8734 | 172512 |
| *Cortinarius* | *austrovenetus* | Cortinariaceae | Agaricales | ectomycorrhizal | 13376 | 39583 | 16006 | 4051 | 8387 | 160123 |
| *Pseudocercospora* | *musae* | Mycosphaerellaceae | Capnodiales | plant_pathogen | 131 | 296 | 695 | 455 | 354 | 159161 |
| *Linnemannia* | *camargensis* | Mortierellaceae | Mortierellales | soil_saprotroph | 16023 | 20411 | 5720 | 10321 | 19029 | 144193 |
| *Amanita* | *flavopantherina* | Amanitaceae | Agaricales | ectomycorrhizal | 143082 | 359990 | 470893 | 40403 | 96416 | 140416 |
| *Russula* | *rugulosa* | Russulaceae | Russulales | ectomycorrhizal | 48641 | 66521 | 216019 | 33475 | 52224 | 124852 |
| *Hebeloma* | *cylindrosporum* | Hymenogastraceae | Agaricales | ectomycorrhizal | 78319 | 25415 | 193647 | 3943 | 5087 | 123937 |
| *Russula* | *vinacea* | Russulaceae | Russulales | ectomycorrhizal | 25921 | 29333 | 403416 | 14003 | 8755 | 118418 |
| *Boletus* | *edulis* | Boletaceae | Boletales | ectomycorrhizal | 18275 | 11553 | 105048 | 6137 | 4845 | 116994 |

**Supplementary Table 4.** The most abundant cDNA contigs encoding CAZymes were assembled from RNA reads from the OS and OM soil layers of fir, spruce, and oak forests.

| **Cazyme family** | ***Abies*_OM** | ***Abies*_OS** | ***Picea*_OM** | ***Picea*_OS** | ***Quercus*_OM** | ***Quercus*_OS** |
| --- | --- | --- | --- | --- | --- | --- |
| **GT2** | 1080 | 563 | 913 | 645 | 1805 | 661 |
| **GT4** | 1013 | 519 | 950 | 586 | 1722 | 581 |
| **GH23** | 580 | 242 | 445 | 208 | 595 | 154 |
| **AA3** | 369 | 259 | 349 | 259 | 537 | 258 |
| **CBM50** | 530 | 323 | 388 | 259 | 529 | 179 |
| **GH15** | 278 | 137 | 285 | 186 | 482 | 196 |
| **CE4** | 374 | 189 | 278 | 200 | 406 | 173 |
| **GT51** | 262 | 90 | 282 | 98 | 405 | 125 |
| **CBM13** | 408 | 489 | 211 | 483 | 347 | 405 |
| **GT35** | 256 | 110 | 192 | 133 | 299 | 105 |
| **GT20** | 226 | 80 | 189 | 83 | 291 | 79 |
| **GH3** | 211 | 123 | 153 | 153 | 289 | 127 |
| **CE11** | 365 | 196 | 309 | 193 | 269 | 87 |
| **GH25** | 305 | 463 | 134 | 380 | 256 | 303 |
| **CBM48** | 235 | 101 | 178 | 107 | 253 | 100 |
| **GH18** | 290 | 330 | 123 | 340 | 212 | 286 |
| **GH13_11** | 117 | 48 | 105 | 53 | 195 | 63 |
| **GT28** | 94 | 29 | 89 | 34 | 188 | 60 |
| **GH74** | 170 | 51 | 185 | 69 | 175 | 40 |
| **GH13_9** | 93 | 27 | 95 | 39 | 167 | 57 |
| **CE14** | 160 | 68 | 166 | 93 | 153 | 88 |
| **GT1** | 105 | 87 | 101 | 92 | 152 | 92 |
| **AA6** | 181 | 311 | 138 | 237 | 148 | 159 |
| **GH38** | 105 | 139 | 74 | 161 | 144 | 200 |
| **GH65** | 95 | 43 | 107 | 55 | 143 | 71 |
| **GH27** | 203 | 272 | 76 | 276 | 137 | 255 |
| **AA5** | 133 | 90 | 73 | 73 | 131 | 39 |
| **GH39** | 60 | 92 | 54 | 91 | 120 | 90 |
| **AA2** | 198 | 289 | 104 | 282 | 118 | 238 |
| **GH17** | 150 | 132 | 115 | 157 | 118 | 98 |
| **GH55** | 76 | 83 | 57 | 83 | 118 | 120 |
| **GH13_16** | 56 | 26 | 68 | 36 | 115 | 50 |
| **GH2** | 102 | 94 | 70 | 111 | 110 | 121 |
| **GH13** | 114 | 52 | 103 | 60 | 106 | 61 |
| **GH16_1** | 134 | 161 | 58 | 147 | 102 | 170 |
| **GH1** | 82 | 47 | 70 | 63 | 101 | 60 |
| **GH176** | 70 | 17 | 80 | 15 | 96 | 13 |

**Supplementary Figures**

**
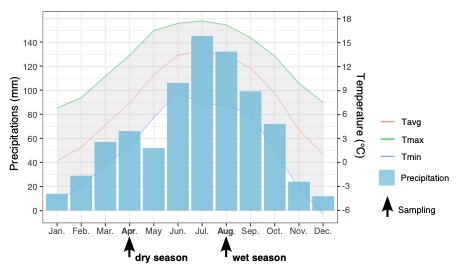
**

**Supplementary Fig. 1.** Annual precipitation and temperature at the Lijiang Forest Biodiversity National Observation and Research Station in Yunshanping, Yulongxueshan, NW Yunnan Province (WorldClim database, <https://www.worldclim.org>). Arrows indicate the sampling periods during the dry and wet (monsoon) seasons.

**Supplementary Fig. 2.** Physicochemical properties of OS and OM soil layers in fir-, spruce-, and oak-dominated forest stands. NS. not significant, * p < 0.05, ** p < 0.01, *** p < 0.001 using the Wilcoxon test.


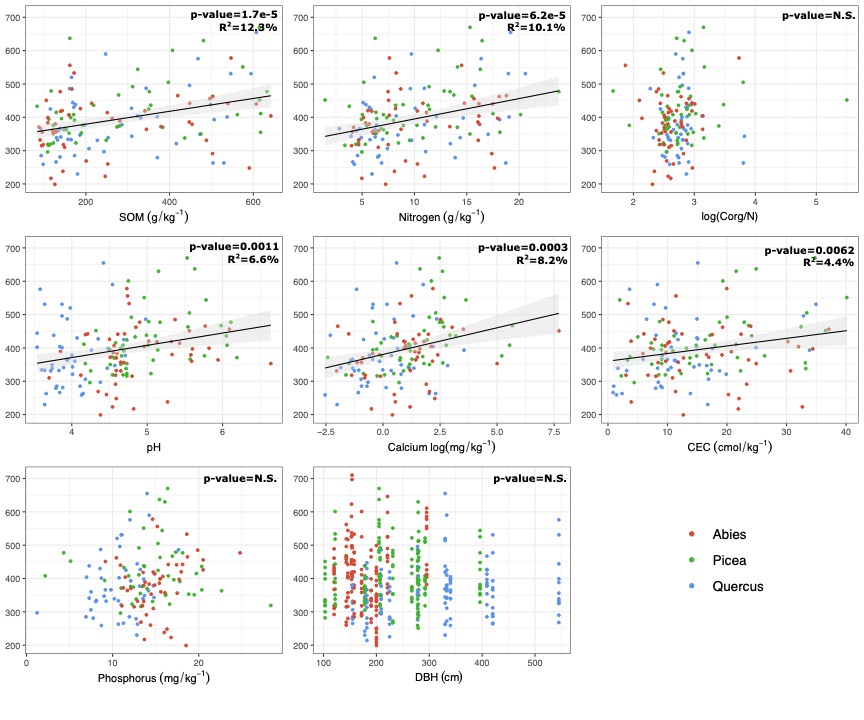


**Supplementary Figure 3.** Correlation between alpha diversity of the soil fungal community, major soil physicochemical characteristics, and tree productivity across the three forest sites. The measured soil features were soil organic matter (SOM), nitrogen (N), Corg/N ratio, pH, calcium, cation exchange capacity (CEC), phosphorus (P), and tree productivity (diameter at breast height [DBH]). The solid lines indicate significant regression lines. The levels of significance and coefficients of determination (R^2^) for all lines are shown in plots with a confidence interval (95%).

**Supplementary Fig. 4.** **Distribution of fungal** **OTUs in soil cores sampled during the dry or wet seasons**. The abundance of OTUs (based on rDNA ITS2) was displayed on a logarithmic scale. OTUs with significant differential abundance were detected using pairwise comparisons with DESeq2 (|LFC| > 5; p.adj < 0.05), followed by forest plots (oak, spruce, and fir) and seasons (dry and wet). Clustering was performed using Ward’s D2 method. Tree species, seasons, fungal phylum and orders, and functional guilds are color-coded as depicted in the right panel. The relative abundance of OTUs is represented by a color scale from the lowest (white) to the highest (dark blue) levels.

**Supplementary Fig. 5.** Fungal transcriptional responses to forest associations and soil layers. Relative abundance (in TPM) of RNA transcripts according to the functional fungal guilds, i.e., ectomycorrhizal fungi (EM), saprotrophic fungi (SAP), plant pathogens and others in the OS and OM layers of the *Abies*-, *Picea*-, or *Quercus*-dominated forests. Green, blue, black and red box plots indicate the abundance of transcripts for ectomycorrhizal, saprotrophic and pathogenic fungi, and other guilds, respectively. Letters represent the results of a TukeyHSD post hoc test of an ANOVA model with a confidence level of 0.95

| **a**  **** | **b**  **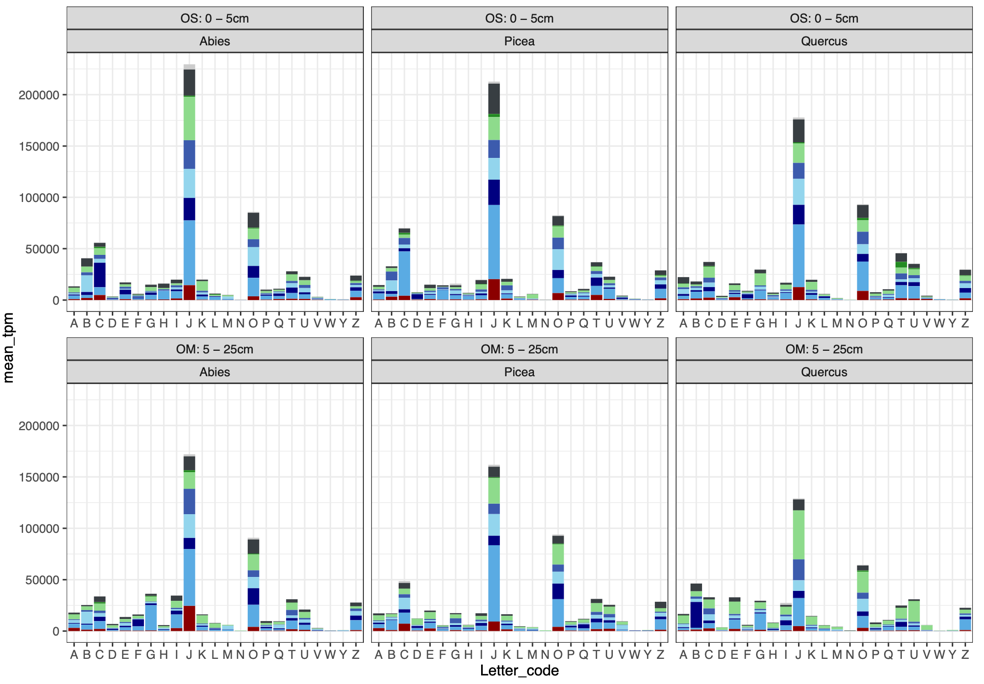**  **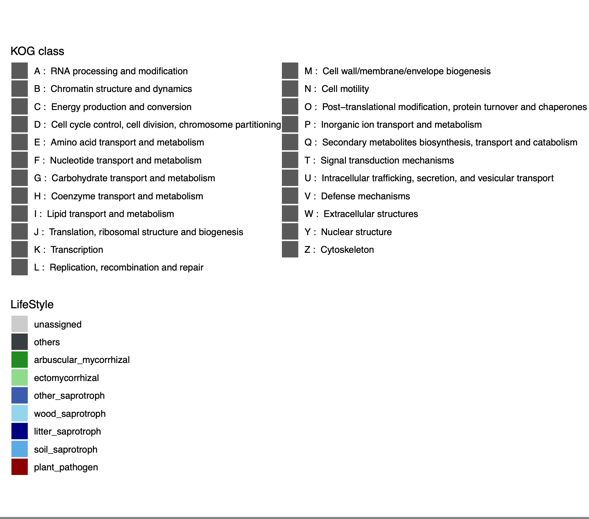** |
| --- | --- |

**Supplementary Fig. 6. Transcriptional responses of genes related to core developmental, cellular, and metabolic pathways in tree species and soil layers.** Distribution of soil fungal transcripts among KOG groups (**a**) and classes (**b**) according to forest associations (oak, spruce, and fir) and soil layers (OM and OS). The bar graphs depict the aggregated sum of transcripts mapped to the genes assigned to the different KOG groups and classes for different fungal lifestyles.

| **a**   | **b**   |
| --- | --- |
| **c**   | **d**   |

**Supplementary Fig. 7. Transcriptional responses of nutrition-related genes to tree species and soil layers.** Heat maps depict an aggregated sum of transcripts mapped to the genes assigned to carbohydrate transporters and assimilation enzymes (**a**), amino acid transporters and assimilation enzymes (**b**), inorganic ion-related transporters and enzymes (**c**), and lipid-related transporters and enzymes (**d**) in soil fungi according to tree species (oak, spruce, and fir) and soil layer (OM, OS). Lifestyles, soil layers, and tree species are color-coded, as indicated in the right panel. The data were clustered using complete linkages, according to similar abundance patterns. The abundance of transcripts (in log scale) is represented by a color scale from the lowest (white) to the highest (black) levels
